# Cortisol Levels of Shelter Dogs in Animal Assisted Interventions in a Prison: An Exploratory Study

**DOI:** 10.3390/ani11020345

**Published:** 2021-01-29

**Authors:** Danila d’Angelo, Serenella d’Ingeo, Francesca Ciani, Michele Visone, Luigi Sacchettino, Luigi Avallone, Angelo Quaranta

**Affiliations:** 1 Department of Veterinary Medicine and Animal Production, University of Naples Federico II, 80137 Naples, Italy; danila.dangelo@unina.it (D.d.); ciani@unina.it (F.C.); sacchettinoluigi@gmail.com (L.S.); avallone@unina.it (L.A.); 2 Animal Physiology and Behavior Unit, Department of Veterinary Medicine, University of Bari Aldo Moro, 70100 Bari, Italy; serenella.dingeo@uniba.it; 3 Dog Park, Ottaviano, 80137 Naples, Italy; m.visone@caniledogpark.com

**Keywords:** Animal Assisted Interventions, welfare, cortisol, physiology, shelter dogs

## Abstract

**Simple Summary:**

Animal Assisted Interventions (AAI) are growing in popularity among professionals of psychological therapies due to their clear benefit for human health. However, to date, little attention has been paid to the welfare of the animals involved in the interventions. In this study we evaluated the potential impact of such activities on the welfare of dogs living in a kennel, which had weekly interactions with inmates held at a prison. To assess their stress levels, we analyzed their physiological response to a stressful situation by measuring the cortisol levels in saliva samples. We found a significant decrease in cortisol concentration at the end of the AAI program measured in the dogs’ living environment (i.e., kennel), which suggests a positive effect of the AAI activities on the welfare of kennel dogs. The results also suggest that transportation from the kennel to the prison could be perceived as a stressful event since it significantly increased dogs’ cortisol levels. Therefore, particular care should be taken in the management of this phase.

**Abstract:**

Previous studies regarding the Animal Assisted Interventions (AAI) have mainly focused on the beneficial effects of human–animal interactions on human health; whereas the impact of such activities on the welfare of the animals involved has received limited attention. So far, few studies have addressed this issue by evaluating the physiological and behavioral reactions of therapy dogs during the interventions. The aim of this study was to evaluate the potential effect of AAI on the cortisol levels of shelter dogs. Five dogs participated in weekly AAI working activities with adult inmates held at a prison of the South of Italy for two months. Saliva samples were collected every two weeks in three conditions: at the kennel (baseline), after transportation and at the end of the working sessions. The results revealed a significant decrease in the cortisol baseline at the end of the AAI program, suggesting that the activities carried out with humans and in a different environment could improve the welfare of dogs housed in kennels. Moreover, we found that transportation significantly increased subjects’ cortisol levels, suggesting that it is a critical phase that deserves particular care.

## 1. Introduction

The development of strong human–animal bonds [1,2] led to an increased inclusion of companion animals in health care and wellbeing services for humans [3,4]. In order to promote research, the Italian Ministry of Health standardized the operational protocols for the Animal Assisted Interventions (AAI) with the “National Guidelines for Assisted Interventions with Animals”, where a therapeutic, rehabilitative, educational and recreational value was attributed to these interventions. They involve the use of domestic animals belonging to the dog, horse, donkey, cat and rabbit species.

The AAI have various and potential benefits for both the people and the animals involved [5,6,7,8,9,10,11,12,13,14,15,16]. Specifically, several studies have reported the positive physiological and psychological effects of AAI for inmates, confirming the general concept that human–animal interactions contribute to human health and welfare [17,18]. Emotions play a key role in human–animal interactions, regulating the expectations and the outcomes of such interactions [19]. Indeed, dogs [20,21,22], as well as cats and horses [23,24], recognize human emotions. Moreover, the emotional valence of previous interactions may affect animals’ reactions in subsequent interactions with humans [25,26]. Therefore, the assessment of emotions during the AAI programs is of crucial importance for the evaluation of animal welfare.

Despite the potential benefits, there are important risks for both animals and humans involved in AAI programs that should not be overlooked, such as the potential transmission of zoonosis [27,28,29,30,31] and the burnout of the animals [32], which is one of the major concerns regarding their welfare. It has been demonstrated that that the growing demand for AAI leads to increased pressure due to more sessions and human users [33]. The decrease in the quality of the interventions can cause uncomfortable situations for the animals involved. This is due to excessive exposure to stressful situations such as the exposure to several unknown environments, the close interaction with unfamiliar people and the unexpected events that could occur during the working sessions [34]. However, the main factor affecting the welfare of the animals involved in the interventions is the poor ability of humans to detect animals’ emotional states that could also lead to human–animal relationship dysfunction [35]. In fact, several authors suggest that children and adults do not clearly perceive dogs’ body signals [36,37]. In particular, children often interpret dogs’ facial expressions of anger as expressing happiness [38]. Indeed, not all emotions may be equally easy to recognize. Overall, people are generally more successful at recognizing positive emotions in dogs, like happiness, while often confusing negative emotions, like fear [37,39,40,41]. Taken together, these factors may contribute to the burnout of animals involved in AAI. Therefore, in the framework of AAI, it is fundamental to evaluate animals’ emotional state during the working sessions with human patients.

Several parameters could be assessed in order to evaluate animals’ emotional state: the variation of physiological (i.e., heart rate, cortisol levels) and behavioral parameters (i.e., stress behaviors) [33,34,42,43]. During stressful situations, the hypothalamus–pituitary–adrenal axis (HPA) is activated, causing the activation of the “fight-or-flight” response (i.e., an increase in alertness and the expression of stress behaviors) and the release of glucocorticoid hormones, including cortisol [42]. Cortisol can be detected in different biological matrices, including urine, saliva and hair [44]. Saliva samples are particularly suitable since they reflect both the activity of the sympathetic nervous system (acute stress) and the HPA [45]. Moreover, the sampling of saliva is a noninvasive method and repetition of the analysis over time allows evaluating the adaptive response even in the medium or long term [46]. Furthermore, the delay in the transfer from blood to saliva (20–30 min) avoid artifacts related to the collecting procedure of samples, which can be a stressful event per se for some dogs.

In the present study we analyzed the variations of salivary cortisol concentrations of shelter dogs involved in an AAI program with inmates. We explored whether the social and environmental enrichment provided by the AAI working sessions could reduce the basal cortisol levels of shelter dogs in their living environment, suggesting an improvement in their welfare. Moreover, we investigated the effect of transportation and AAI activities on the cortisol concentration of the tested dogs in order to evaluate the potential effect of these events on the dogs’ physiological stress response. We hypothesized that the enrichment provided by the AAI activities could reduce the basal level of cortisol of shelter dogs and that, on the other hand, transportation could increase cortisol concentration, eliciting a state of excitement in dogs.

## 2. Materials and Methods

### 2.1. Participants

Five dogs were involved in the study. They were mixed breed, both males (*n* = 3) and females (*n* = 2), all neutered, of various sizes: small (*n* = 1), medium (*n* = 2) and large (*n* = 2) (based on classification reported by Wilding [47] (see Table 1). Their age ranged from 2 to 3 years (2.4 years ± 0.55; mean ± S.D.). They had been housed in the kennel for at least 16 months (18.8 ± 3.35; mean ± S.D.) and never underwent any basic educational training before the beginning of the study.

They were selected from the guests of the Coop kennel, Dog Park of Ottaviano (Naples), where they were housed after being caught on the territory by the local health authority, in order to limit the stray phenomenon. The structure of the Coop kennel is organized in multiple boxes to meet the social needs of the canine species. Each box presents a surface of 6 m^2^/dog with an open space in front of it, where dogs could have a walk and interact with each other.

The subjects were selected by a veterinary behaviorist using the evaluation scale for emotional disturbs of dogs (EDED Scale) of Pageat [48] (Supplementary Materials Table S1), which allows the classification of dogs’ behavior according to the presence/absence of centripetal and centrifugal behavior and the expression of homeostasis or emotional disturbances. The centripetal activities are represented by feeding, drinking, self-directed behaviors and sleep; while the centrifugal activities are social contacts, exploratory capacity and aggression. For each behavior considered, a specific score was attributed to each subject. Each dog then obtained a total score, which indicated its general emotional state (see Supplementary Materials Table S2 for score range and corresponding emotional state). The dogs selected had a score from 9 to 12, corresponding to a normal state for emotional and cognitive profile. This value is attributed to dogs with an adequate prosocial profile suitable for the AAI activities (the individuals that obtained a score <9 and >12 were excluded). Therefore, from an initial population of 25 subjects, we selected 5 dogs that participated to the study.

Before taking part in the study, the dogs undertook a clinical visit and laboratory tests (complete blood count, clinical biochemistry evaluation) to certify their health. Moreover, a veterinary behaviorist certified the absence of any behavioral pathologies. Moreover, the selected dogs were grouped together in a box from one month before the beginning of the tests until the end of the study. This procedure allowed the creation of positive relationships between the dogs and the avoidance of agonistic interactions (including aggression and fear responses) during the AAI activities with inmates.

### 2.2. Procedures

The AAI activities were carried out in the outdoor area of the prison. The AAI sessions were conducted on the basis of the Italian National Guidelines for Animal-Assisted Interventions [49], which required the presence of a veterinary certified expert in AAI, a project manager, an intervention contact and dog assistants. The recruited operators had all attended the courses for dogs assistants required by the aforementioned national guidelines.

The dogs were transported to the prison in individual steel cages (97 × 90 × 70 cm) placed in a van which met the requirements established by Italian law in terms of the number of individuals, space for each subject and ventilation. Two people (the driver and a person taking care of dogs) were in the vehicle during the transportation, which had an average duration of 1 h (70 km from the kennel to the prison).

The AAI activities were carried out weekly during a period of two months. In each session, each dog worked with two inmates. A total of 10 inmates (all males, whose age ranged between 31 and 40 years) took part to the study. The activities were supervised by an animal assistant (who actively intervened for the safeguarding of the proper management of the animal during the interaction and of its state of well-being, according to the criteria established by the veterinarian) and by the intervention contact (who guided the person during the sessions). The total duration of each session was approximately 70 min: 10 min were dedicated to the transfer of the theoretical part to the prisoners, in which the dogs were left free to explore the environment and interact with each other; 60 min for the practical interaction between dogs and inmates (equally divided between the two inmates interacting with the same dog), with a break of 20 min after 30 min of working to safeguard animal welfare [50].

The proposed activities for the human–dog interactions were:

(1) How and when to pet the dog,
(2) Luring technique (to capture the dogs’ attention),
(3) Turn to the inmate’s right or left,
(4) The command “sit”,
(5) Nose-working activity,
(6) The command “stay”,
(7) Management of the leash,
(8) Recall,
(9) The command “give the paw”.

At least two activities were carried out in each session, following the list illustrated above (all activities were carried out at least once). The training was performed using positive reinforcements (treats and vocal reinforcements) and gentle management (no physical or psychological pressure was induced) using the luring technique.

### 2.3. Sample Collection and Cortisol Measurement in Dogs

The dogs’ physiological stress responses were monitored during the entire project through the measurement of salivary cortisol, which accurately reflects the blood changes of this hormone [51] with a delay of 20–30 min [44,45,52]. Therefore, it was used as a measure of the acute response to stress, evaluating the adaptive response of dogs as immediate reactions to the interaction with the inmates.

The dogs had been previously accustomed to the procedure described by Salivette Cortisol (Stastedt, AG & Co., Numbercht, Germany) for saliva collection to determine salivary cortisol concentrations. Commercial biocompatible synthetic fiber rolls in tubes (Salivette^®^, Sarstedt, AG & Co., Numbercht, Germany) were used to collect saliva samples. From a month before the beginning of the study, once a week a salivette was placed into each dog’s mouth for a minute. The increase in salivation was facilitated by the olfactory perception of the dogs’ favorite food. Moreover, the subjects were not allowed to drink and eat in the 30 min preceding the collection of the samples in order to register reliable cortisol values [49]. The dogs had free access to water during the AAI working sessions. At the end of the activity, the water bowls, as well as the food treats used during the training were removed (30 min before the saliva sampling).

During the testing period, three salivary samples were taken for each working session. The first sampling was carried out at 8.20 a.m., before the departure from the kennel (T0). The second one was taken upon arrival at the prison around 9.30 a.m. (T1); the third sample was obtained at the end of the AAI activity, around 12.30 p.m. (30 min after the end of the session, T2). Saliva samples were collected every two weeks (3 samples × session) for a total of 5 different working sessions (D1, D2, D3, D4, D5). Therefore, a total of 15 saliva samples were obtained for each subject.

The collected samples were placed in a polystyrene container with ice and transported to the laboratory, where they were promptly centrifuged at 3000 rpm in a refrigerated centrifuge for 15 min to obtain the saliva for cortisol determination. The samples then obtained were stored at −20 °C and then proceeded. Cortisol was determined by immunoassay using the commercially available Salivary Cortisol kit (Salimetrics, State College, PA, USA) according to the manufacturer’s indications.

### 2.4. Data Analysis

In order to evaluate the salivary cortisol level before, during and after the AAI activities, the statistical analysis was performed using SPSS software. Data distribution was tested using Shapiro–Wilk test. Wilcoxon signed rank test was used to analyze the differences of the dogs’ cortisol levels registered during the experimental period, and particularly before the beginning of each working day (T0, baseline), after the transportation (T1) and at the end of each working session (T2). Results were considered statistically significant for *p* < 0.05.

## 3. Results

### Dog Salivary Cortisol Levels

**Figure 1:**
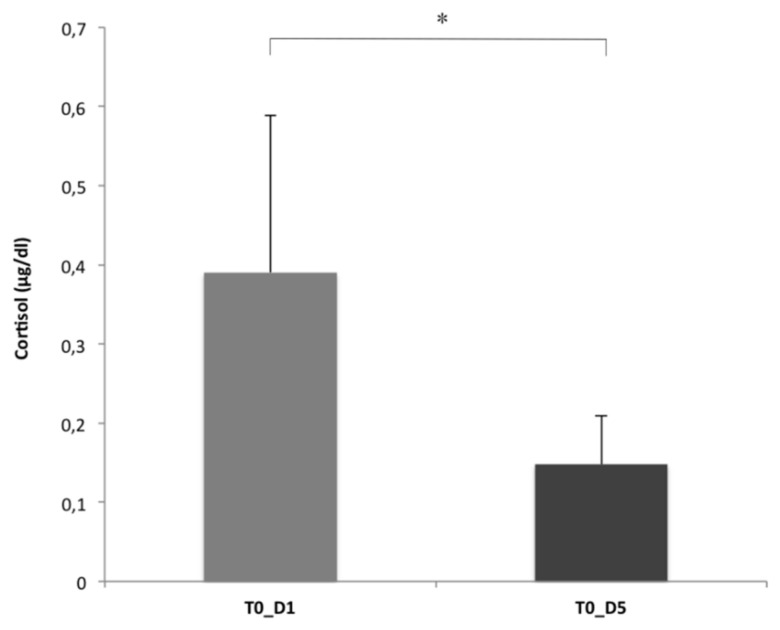
Cortisol concentration in the saliva samples collected at T0 on D1 and D5 (means and S.E.M. are shown; Wilcoxon signed rank test). * *p* < 0.05.

The statistical analysis revealed a significant decrease in the dogs’ cortisol basal levels at the end of the AAI program. Specifically, the cortisol concentration in saliva samples collected in the kennel before the beginning of the AAI program (T0 at D1) was significantly higher than the one registered at the last working day, within the same context (T0 at D5) (*n* = 5, Z = 0.00, *p* = 0.043; Wilcoxon signed rank test) (Figure 1).

**Figure 2:**
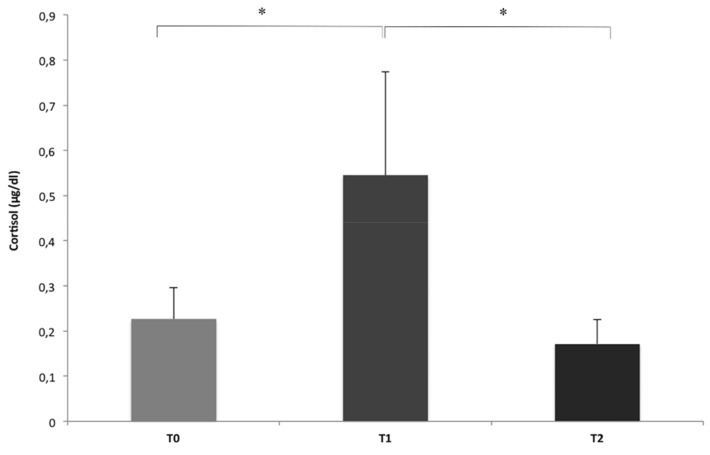
Differences in the mean cortisol levels of dogs collected at T0 (baseline, at the kennel), T1 (after transportation) and T2 (end of AAI sessions) of the five working sessions (means and S.E.M. are shown; Wilcoxon signed rank test). * *p* < 0.05.

Furthermore, results showed that the dogs’ cortisol levels were generally and significantly higher after the transportation (T1) than those registered at the kennel (T0, baseline) and at the end of the working sessions (T2) (T0 vs. T1: *n* = 5, Z = 15.00, *p* = 0.043; T1 vs. T2: *n* = 5, Z = 0.00, *p* = 0.043; Wilcoxon signed rank test). No statistically significant differences were found between the cortisol levels observed at the kennel and at the end of the working sessions (T0 vs. T2: *n* = 5, Z = 1.00, *p* = 0.080; Wilcoxon signed rank test) (Figure 2).

## 4. Discussion

In the present study we provide preliminary evidence for the potential effectiveness of human–animal interactions in the framework of Animal Assisted Intervention for the improvement of the general welfare of kennel dogs. Our results showed, indeed, a significant decrease in the cortisol basal levels sampled at the kennel (T0) on the last working day (D5) (and before the last working sessions) with respect to those registered before the beginning of the AAI program (D1). Moreover, we observe no significant differences in the salivary cortisol collected at the kennel (baseline, T0) and at the end of the working sessions (T2) on the five working days. These findings suggest that the working activities with inmates could have not been perceived as a stressful event for dogs but, most importantly, it could have produced clear benefits for them, reducing the animals’ general stress levels registered in their living environment (the kennel) and, therefore, improving their welfare.

Our results are consistent with previous studies observing that the interaction with humans in a different environment than the living environment (i.e., an isolated room in the shelter [53] or at a private home [54]) cause a decline in cortisol concentration of dogs housed in kennels. It has been found that the mere presence of a person, in particular the dogs’ caretaker [55] or an immobile, noninteractive woman [53], positively impacts the physiological stress response of shelter dogs, reducing their cortisol levels. Similar results have been registered after human–dog interactions but their efficiency is strictly dependent on the nature of such interactions. Specifically, a gentle petting [56,57] and a play session mainly based on affiliative behaviors [58] are effective for the cortisol decrease. However, this effect seems to be temporary; the cortisol concentration returns to the basal level when the animals are brought back to the shelter [59]. On the contrary, in the present study we registered a significant decrease in the saliva cortisol baseline at the end of the AAI program, suggesting that the working activities with humans might have a long-term impact on the general stress levels of kennel dogs, not only a short-term effect measured at the end of each session. In other words, the social as well as the environmental enrichment provided by the frequent interactions with humans in a different environment (which became familiar) than the living environment could positively impact the emotional and affective state of dogs housed in a kennel, improving their welfare. However, the predictability of the environment where the AAI program takes place is a crucial factor since undertaking the activity in a novel environment could produce, instead, an increase in cortisol levels in the tested subjects [60].

Previous studies reported that the decrease in cortisol levels of kennel dogs across time could be also related to the animals’ habituation to the living environment. This phenomenon generally produces the return to basal level within 10 days after the arrival but not later than the first month [61,62]. As a consequence, it is unlikely that the significant decline of the cortisol basal concentration here reported could be related to the habituation to the kennel environment since dogs involved in the program had already been living in the shelter for at least 16 months. Moreover, the selected dogs were grouped together one month before the beginning of the tests, in order to avoid the eventual acute stress due to the novel living conditions impacting their basal cortisol levels. Another possible explanation for the general decrease in cortisol levels in kennel dogs could be related to the dysregulation of the HPA-axis due to chronic stress reported by some authors [61]. However, this issue is still a matter of ongoing discussion and further data are needed to support this hypothesis. In the present study, since the subjects involved in the program were selected considering their normal state for emotional and cognitive profile, the latter hypothesis remain an unlikely prospect.

On the other hand, the lack of significant difference between the cortisol levels measured at the kennel (baseline) and at the end of the working sessions could further support the hypothesis that the AAI activities did not produce a physiological stress response in the shelter dogs. This finding is consistent with previous studies reporting no differences in the cortisol levels between pre- and post-session in therapy and pet dogs [63]. Sandri and colleagues [64] have also registered a significant decrease in dogs’ salivary cortisol concentrations 20 min after the end of the AAI session compared to those measured before the start. However, these authors have argued that the lack of difference in the cortisol concentration observed before and after the interactions with humans might be an outcome of the animals’ adaptation to both the social and physical environment where the sessions take place [64]. Therefore, one possible explanation for the lack of differences in the cortisol levels found between the baseline and the measurement performed at the end of the working session might be related to this phenomenon.

Another interesting aspect to consider is that the transportation has a significant impact on the cortisol levels of the dogs involved in the AAI programs. The salivary cortisol values registered upon the arrival to the prison where the working sessions took place were higher than those registered in the kennel and after the end of the sessions for the total collected samples, suggesting that transportation may constitute a critical phase that needs to receive further and deep consideration. One possible explanation for the marked increase in the salivary cortisol after transportation is that the dogs could have perceived this event as the most stressful. The limited space for each dog, the restraint as well as the social confinement and the long duration of the journey (70 km from the kennel to the prison) are the potential factors causing the increase in salivary cortisol found here. This hypothesis is supported by evidence coming from studies on farm animals (horses [65], cattle [66,67], pigs [68]) as well as dogs [69] and mice [70], showing that transportation cause a physiological stress response that is reflected in the increase in cortisol concentrations in different samples (blood [66,68,69,70], saliva [65,68] and feces [65,67]). In these studies, the combined evaluation with other physiological (e.g., changes in heart rate and heart rate variability [65], glucose [66,69], cholesterol and triglyceride [69]) and behavioral parameters [68,70] suggested that transportation could be a crucial and stressful event, which could negatively impact these species’ welfare. In the light of this, appropriate and specific measures should be adopted that could reduce, for instance, the duration of the journey and improve the transportation conditions (spaces, ventilation, temperature etc.) [41]. Animals should be previously habituated to lie in a travel box, than to get on and off the vehicle and to the general movement of the car or van, since the repetition of transport procedures seems to be effective for the decrease in the animals’ stress responses due to their habituation [71]. AAI protocols should also include an initial phase before the beginning of the working session with patients where dogs could recover after the transportation, having time for a relaxed walk and to explore the settings in order to reach a neutral emotional state. It could be also profitable to arrange activities within a structured facility hosting dogs and receiving patients for the AAI programs for suitable activities.

Since the HPA-axis activation expresses the increase in arousal which is independent from the valence of the emotion experienced [72,73], we cannot entirely rule out the possibility that the high levels of salivary cortisol registered in response to transportation reflect a state of general excitement due to the anticipation of a positive event/outcome (i.e., the different environment or the interaction with inmates) [60]. Therefore, given that cortisol could be secreted both during positive and negative affective states, further investigations are required for correctly interpreting our findings and the impact they could have on animal welfare.

In our study, five subjects took part in the program. This specific number derives from the selection of suitable individuals from an initial population of 25 subjects according to their sociability, docility and ability to communicate with both conspecifics and humans, which are all fundamental characteristics for the AAI activities.

Although our study considers only salivary cortisol as a physiological biomarker for measuring animal welfare, our results open the doors for further investigation about the benefit of AAI for the improvement of the welfare of kennel dogs. Besides the evaluation of animal behavior [34], a comprehensive assessment of animal welfare based on easy-to-detect physiological measures (i.e., by noninvasive procedures that could be stressful by themselves) could include the evaluation of β-endorphin [74], salivary Arginine vasopressin (AVP) [75] and IgA [76] concentrations, vanillylmandelic acid/creatinine ratios (VMA/Cr) in urine [77], specific biochemical parameters (e.g., acute phase and glycated proteins [74]) as well as each dog’s surface temperature (measured by infrared thermography [77,78]), which have been demonstrated to be reliable measures of animal stress levels.

## 5. Conclusions

Overall, our results showed a significant decrease in the shelter dogs’ basal cortisol levels measured in the saliva at the end of the AAI program with respect to the cortisol concentration registered before the beginning of the experimental period. This finding suggests that the interactions with humans and the activities carried out in a different environment could improve the welfare of dogs housed in kennels. Moreover, we highlight the importance of transportation as a critical event since it significantly increases the subjects’ cortisol levels, suggesting that particular care should be taken in the management of this phase. Our results open the door for further investigations of possible and potential benefits of AAI activities for kennel dogs.

## Figures and Tables

**Table 1:** Characteristics of dogs included in the Animal Assisted Interventions (AAI).

Subjects	Sex	Age (Years)	Size	Time Spent in the Kennel (Months)
Gigio	Male	3	Small	20
Ketty	Female	2	Medium	16
Sara	Female	2	Large	18
Lupetto	Male	2	Medium	16
Lean	Female	3	Large	24

## Data Availability

Not applicable.

## Supplementary Materials

The following are available online at https://www.mdpi.com/2076-2615/11/2/345/s1, Table S1: Evaluation of Dogs’ Emotional and Cognitive Disorders (EDED Scale), Table S2: Score values referred to EDED Scale.
