# Supplementary material for: Cortisol Levels of Shelter Dogs in Animal Assisted Interventions in a Prison: An Exploratory Study
Source: Animals (Basel). 2021 Jan 29;11(2):345. doi: 10.3390/ani11020345 (PMC7911336; doi:10.3390/ani11020345)
Supplement: Supplementary file 1 [file animals-11-00345-s001.pdf]

Cortisol Levels of Shelter Dogs in Animal Assisted Interventions in a Prison: An Exploratory Study

Danila d'Angelo, Serenella d'Ingeo, Francesca Ciani, Michele Visone, Luigi Sacchettino, Luigi Avallone and Angelo Quaranta*

*Correspondence: angelo.quaranta@uniba.it; Tel.: +39-080-544-3927

Table S1. Evaluation of Dog's Emotional and Cognitive Disorders (EDED Scale).

Behavior type	Specific behavior	Score
**Centripetal**		
Feeding	Normal appetite	1
	Hyperphagia (with regurgitation and reingestion)[1]	3
	Anorexia/hyporexia	4
	Dysorexia (moving from hyper to hypo)	5
Drinking	Normal drinking	1
	Carries empty water bowl around (ritual)[2]	2
	Chews at water without swallowing[3]	3
	High-frequency drinking (documented)	5
Self-stimulatory	Normal cleaning behavior	1
	Excessive licking, nibbling[4]	4
	Stereotyped nibbling, dizziness, turning on itself (or other stereotypies)[5]	5
Sleep	Normal (or no change)	1
	Increase in sleep, hypersomnia[6]	2
	Insomnia, during sleep (and hyposomnia)[7]	3
	Wakes up shortly after falling asleep, anxiety at time of going to sleep (and restlessness)[illegible]	5
**Centrifugal**		
Exploratory (scanning)	Normal	1
	Inhibited	2
	Frequent avoidance responses	3
	Increased, hypervigilant	4
	Oral	5
Aggression (defense)	No aggression or aggression stable (no increase or decrease)	1
	Irritation-related aggression	3
	Fear-related aggression	4
	Displays both fear and irritation aggression	5
Learned social behavior	Unchanged	1
	No submission response	2
	No self-control when playing	2
	Bites without growling	4
	Steals, does not drop stolen objects	5
Specific learned behavior	Same response capacity (allowing for disease or age)	1
	Arbitrary responses	3
	No response to previously learned behaviors	5
Physical exam[9]	Normal	1
	Periods of tachycardia and/or tachypnea	2
	Diarrhea, colic	2
	Dyspepsia (and ptyalism)	2
	Increased emotional micturition	3
	Acral lick granuloma (and extensive lick alopecia)	4

Table S2. Score values referred to EDED Scale.

E.S.E.D. value	Interpretation
9-12	Normal state
13-16	Phobias
17-35	Anxieties
36-44	Emotional (Thymic) disorders
